# Neutrophils and Close Relatives in the Hypoxic Environment of the Tuberculous Granuloma: New Avenues for Host-Directed Therapies?

**DOI:** 10.3389/fimmu.2019.00417

**Published:** 2019-03-12

**Authors:** Aude Remot, Emilie Doz, Nathalie Winter

**Affiliations:** INRA, Universite de Tours, UMR Infectiologie et Sante Publique, Nouzilly, France

**Keywords:** neutrophils, *Mycobacterium tuberculosis*, granuloma, lung, HIF, hypoxia, host-directed therapies

## Abstract

Tuberculosis (TB), caused by *Mycobacterium tuberculosis* (Mtb) is one of the most prevalent lung infections of humans and kills ~1.7 million people each year. TB pathophysiology is complex with a central role played by granuloma where a delicate balance takes place to both constrain bacilli and prevent excessive inflammation that may destroy lung functions. Neutrophils reach the lung in waves following first encounter with bacilli and contribute both to early Mtb elimination and late deleterious inflammation. The hypoxic milieu where cells and bacilli cohabit inside the granuloma favors metabolism changes and the impact on TB infection needs to be more thoroughly understood. At the cellular level while the key role of the alveolar macrophage has long been established, behavior of neutrophils in the hypoxic granuloma remains poorly explored. This review will bring to the front new questions that are now emerging regarding neutrophils activity in TB. Are different neutrophil subsets involved in Mtb infection and how? How do neutrophils and close relatives contribute to shaping the granuloma immune environment? What is the role of hypoxia and hypoxia induced factors inside granuloma on neutrophil fate and functions and TB pathophysiology? Addressing these questions is key to the development of innovative host-directed therapies to fight TB.

## Introduction

Tuberculosis caused by *Mycobacterium tuberculosis* (Mtb) is present worldwide. With estimated 10.4 million new cases and 1.7 million deaths in 2016^1^, TB remains one of the most devastating respiratory disease of human kind. The key cell in Mtb lung infection is the lung alveolar macrophage (AM) that engulfs the bacilli and orchestrates the adaptive host immune response if bacilli are not eliminated (1). This is the starting point for the granuloma, set as an immune defense mechanism that eventually becomes the pathologic signature of Mtb infection. AM plays major roles in the battle between Mtb and the host and a large body of the literature is devoted to this key cell. However, mature neutrophils circulate in high numbers in blood and are also sequestered in the lung (2). As they are present in the early phase of Mtb infection, before the onset of adaptive immunity, they could play important beneficial protective roles [see extensive review in (3)]. In the zebra-fish (ZF) embryo infected with *M. marinum* (Mm) as a surrogate of Mtb infection in mammals, neutrophils come in response to signals sent by Mm-infected dying macrophages (MPs). Neutrophils dispose off Mm-infected MPs by efferocytosis in the nascent granuloma and kill bacilli through NADPH oxidase-dependent mechanisms (4). We and others have shown in resistant mouse models that neutrophils come in two different waves after Mtb infection before and after the onset of adaptive immunity (5, 6). While the first wave was found to phagocytose BCG–the vaccine strain used against Mtb–*in situ* in the lung, the second T-cell dependent wave was seldom associated with bacilli. In response to virulent Mtb, T-cell dependent neutrophils did not control Mtb growth but rather established close contacts with T-cells in the granuloma (6) suggesting their role in regulation of the adaptive immune response. This is in line with their established role in the formation of the structured mature granuloma in mice (7). However, during active TB, it is now consensus that neutrophils are largely responsible for lung destruction (8). They are the most represented cell subset in sputum (9) and drive an interferon-inducible transcriptional signature in blood cells during active TB (10). Several excellent reviews recently covered neutrophils as “good and bad guys” during TB (3, 8, 11, 12). Such opposite roles may depend on several factors including timing and magnitude of neutrophil recruitment or different neutrophils subsets which respective roles in TB remain elusive. Despite the fact that neutrophils are established as key players in the TB granuloma, the impact of hypoxia on their behavior and functions is still poorly explored and we advocate in this review that this should be reconsidered. Moreover, in the granuloma, the influence of the hypoxic milieu on contribution of neutrophils to production of soluble mediators involved in TB pathophysiology needs to be reconsidered. The world is on high demand of host-directed therapies (HDTs) as adjunct to antibiotics to fight against TB and we hope that our mini review will help to design effective strategies by taking into account the impact of hypoxia on neutrophils.

## The Mtb Granuloma is a Pathological Immunogical Niche Where Neutrophils Play Major Role

For a long time, the granuloma has been considered as an uniquely host-driven response, set to constrain Mtb and prevent bacilli dissemination. This view was challenged when, in ZF embryo, virulent Mm was shown to disseminate and establish infection by manipulation of the nascent granuloma and adjacent stromal cells (13). Today, the host-pathogen mutual benefit of the granuloma is still a matter of debate (14, 15), as is the role of neutrophils in this structure. Some confounding interpretations may come from animal models, especially the mouse, most extensively used in TB research. In humans, primary TB leads to caseating granulomas that necrotize over time. Cavities, allowing Mtb transmission, represent the most severe signature of the disease (16). Human TB granulomas are hypoxic as demonstrated by Positron Emission Tomography-Computed Tomography (PET-CT) scans using hypoxia-specific tracers in patients with active TB (17). In TB susceptible animals such as the rabbit, the guinea pig, and the non-human primate, hypoxic granulomatous lesions develop in the lung (18). By contrast, the resistant mouse lines C57BL/6 and BALB/c that have been extensively used, do not develop necrotizing hypoxic granulomas which brought the quite general belief that mice are not an adequate model for TB pathophysiology studies (19). However, extremely susceptible mice such as C3HeB/FeJ do develop central caseous necrosis in the lung (20) and these lesions are hypoxic (21). A common feature to all TB susceptible animals that develop hypoxic necrotizing granulomas is the abundance of neutrophils (22, 23). Mtb induces necrosis of human neutrophils, which depends on its main virulence factor, the small protein ESAT-6 secreted by Type VII secretion system. Necrosis is driven by neutrophil-derived Reactive Oxygen Species (ROS) and is required for Mtb growth after uptake of infected neutrophils by human macrophages (24). In C3HeB/FeJ mice, neutrophils dying by necrosis or NETosis rather than apoptosis seem to drive the caseous necrosis and liquefaction process (25). The crucial role of neutrophils and the S100A9 inflammatory protein for granuloma formation is demonstrated (26). Therefore, what “adequate” animal models and available pathology studies in humans teach us is that neutrophils and hypoxia are crucial to the development of lung lesions during TB disease.

However, some clarification is needed regarding the definition of neutrophils. These cells have long been considered as an homogenous population based on their polylobed nucleus and a minimal set of markers: in mice, they are defined by flow cytometry as Gr1, CD11b double positive cells or more recently as CD11b, Ly-6C, Ly-6G triple positive cells. In humans, they are still minimally identified as CD11b^+^ CD14^−^CD15^+^ cells. The picture has become more complex with the description of Myeloid Derived Supressor Cell (MDSCs), which largely share markers with neutrophils. MDSCs are an immature and heterogeneous population present at homeostasis and accumulating in pathological situations. Originally described in cancers, MDSCs are increasingly characterized in inflammatory diseases (27, 28). MDSCs suppress T cell responses, via different mechanisms, including production of ROS, nitric oxide (NO), or arginase 1 (29). MDSCs are present as two main subsets: monocytic MDSCs and granulocytic MDSCs (Gr-MDSCs). The later display the same morphology and phenotype as *bona fide* neutrophils. They share the Ly-6G or Gr1 markers. Therefore, MDSCs can robustly be distinguished from *bona fide* neutrophils only based on their suppressive function (30). Expansion of granulocytic and monocytic MDSCs is observed in blood of active TB patients and healthy recently exposed contacts (31, 32). This correlates with enhanced L-arginine catabolism and NO production in plasma from active TB patients (33). In resistant (C57BL/6) or susceptible (129S2) strains MDSCs–defined as Gr1^+^ cells–are identified in the lung parenchyma during the course of Mtb infection where they suppress T cells (34). They also vigorously ingest Mtb. Interestingly, in susceptible mice, Gr1^+^ MDSCs cells accumulate in higher numbers and phagocytoze more bacilli as compared to resistant mice. Therefore, MDSCs could represent a niche for Mtb replication, helping the pathogen to escape the immune system (34). MDSCs are also associated with TB progression and lethality (35). These findings emphasize the potential of MDSCs as targets for immunotherapy. However, most studies using depletion antibodies that target the Gr1 or the Ly-6G surface marker, do not allow today a clear distinction of the role of *bona fide* neutrophils vs. MDSCs in TB pathophysiology. To add to the complexity, the low density neutrophils (LDNs) have recently been described as a new population of neutrophils. LDNs, displaying heterogeneous morphology and containing mature and immature cells, are immunosuppressive via secretion of IL-10 and expression of arginase-1 (36). Interestingly, mature high density neutrophils (HDNs) can switch to LDNs in a TGF-β dependent way, and acquire immunosuppressive functions similar to granulocytic MDSCs (37). First described in cancer (37) and pulmonary pathologies (38), LDNs have also been identified in TB and associated to the severity of the disease. Moreover, Mtb is able to convert HDNs to LDNs *in vitro*, suggesting manipulation by Mtb (39). Even though LDNs are not yet considered as granulocytic MDSCs, the largely shared purification procedure, analysis methods and markers between these two cell populations suggest possible overlaps (30). Mtb infection in mice recruits an altered “neutrophil” population defined as “Gr-1^int^/Ly-6G^int^” cells with lower levels of Gr1/Ly-6G as compared to classical neutrophils. These immature cells highly express the CD115 and CD135 markers and inhibit T cell proliferation (35). Whether these cells are distinct from granulocytic MDSCs remains to be clarified.

Outside from the TB research field, recent studies identified neutrophils as potential players in inflammatory angiogenesis. Neutrophils store Vascular Endothelial Growth Factor (VEGF), a key player in the process of angiogenesis, that they may release upon stimulation. By recruiting neutrophils, MIP-1α and MIP-2 act in an autocrine loop to promote this process. A new CXCR4^high^ and CD49d^high^ neutrophil subset, displays angiogenic properties via secretion of high concentrations of MMP-9 promoting neovascularization (40). In a model of skin hypersensitization, Tan et al. demonstrated that neutrophil MMP9 and heparanase, targeting distinct domains of the extracellular matrix, cooperate to release diverse VEGF isoforms and influence their bioavailability and bioactivity during inflammatory angiogenesis (41). In mice and humans, CD49d^+^ CXCR4^high^ VEGFR1^high^ neutrophils are recruited specifically in hypoxic ischemic tissues in a VEGFR1 and VEGFR2 dependent way (42). Whether such neutrophils could contribute to formation of the hypoxic TB granuloma remains on open question.

## Hypoxia-Induced-Factors are Master Regulators in Mtb Granuloma

The tremendously exciting field of immunometabolism, which links cellular bioenergetics pathways to immune cell functions, brings new views on the fate of the TB granuloma. In response to inflammatory environment, MPs switch from oxidative phosphorylation–the mitochondrial respiration system that quiescent cells use to generate energy–to aerobic glycolysis. This shift, called the Warburg effect, was discovered in proliferating cancer cells that highly incorporate glucose, that they convert to lactate while producing ATP and cell-building blocks (43). Master regulators of this switch are Hypoxia-Induced–Factors (HIFs), a family of transcription factors that govern cell reprogrammation (44). Under normoxia, the enzymes Prolyl Hydroxylase Domains (PHD) and Factor Inhibiting HIF (FIH) repress HIFs via targeted degradation and transcriptional mechanisms. Under low O_2_ tension, these enzymes become inactive, HIFs are stabilized and derepressed and activate a transcriptional program to adapt the cell to hypoxia. Other than O_2_, HIFs respond to a variety of environmental factors. HIF1α^−/−^ mice display enhanced Mtb burden and reduced survival (45). This could be linked to HIFs regulating the bactericidal functions of MPs and neutrophils (46). NF–kb, the master regulator of the inflammatory response, regulates transcription of the *hif1a* gene encoding one of the HIF subunits (47). In MPs, LPS binding to TLR4 activates HIF-1α that upregulates IL-1β production. The signaling occurs through succinate, one intermediate of the tricarboxylic acid cycle (48) that accumulates upon reprogrammation of the MP toward aerobic glycolysis. However, this effect cannot be generalized to all TLR- signaling pathways (49). Imaging with glucose tracers illustrates high glucose uptake after infection with Mtb in the lungs of C3HeB/FeJ mice (50) non-human primates (51) and humans (52). Aerobic glycolysis is confirmed by NMR-analysis of metabolites in mice (53) and guinea pigs (54), or global transcriptomics of genes encoding glycolytic enzymes in the lungs of mice, rabbits, and humans (55). Reprogramming of the host metabolism translates in coordinated up and down regulation of genes encoding key glycolytic enzymes and glucose transporters, reminiscent of the Warburg effect as well as regulation of HIF-1α (55). While the granuloma becomes necrotic, MPs packed with lipid droplets transform into foamy cells (56) which is driven by reprogrammation of host lipid metabolism in response to Mtb compounds (57). Interestingly, lipid droplets formation in Mtb infected MPs is driven by IFN-γ and requires HIF-1α and its target gene *hig2* (58).

Several immunopathology studies demonstrate extensive vascularization of TB granulomas in humans (59) and mice (59, 60) provided that they are not necrotic (61). The link between hypoxia, vascularization, and development of the granuloma was recently established in the ZF infected with Mm (62). In this model, HIF-1α is activated, PHD-3 expression is increased and induces production of the angiogenic factor VGEF-A, which is intimately linked to nascent granuloma formation. In human MPs infected with Mtb, a similar angiogienic signature is observed (63). Moreover, the level of VEGF-A is increased in sputum and peripheral blood of active TB patients and is proposed as a differentiating biomarker for patients progressing to active TB (64–66). Circulating angiogenic factors are markers of disease severity and are associated to the bacterial burden (67). In ZF embryos infected by Mm, CXCR4 signaling is important to initiate angiogenesis for granuloma expansion (68). Surprisingly, despite the established over-representation of neutrophils in TB lesions, little information is available on how these cells behave in face of Mtb in the highly hypoxic and angiogenic granuloma milieu.

Neutrophils are generally seen as short-lived cells. However, the life span of neutrophils is highly increased in hypoxic milieu (69). By high consumption of oxygen during oxidative burst, neutrophils themselves contribute to generate the hypoxic milieu, which may well be the case during active TB when they invade the lung. Prolonged survival is linked to sustained expression of PHD3, *in vitro* and *in vivo*, in response to hypoxia and inflammatory stimuli (70). Interestingly, PHD3 is strongly induced in lungs of Mtb infected mice (55). HIF-2α is the most expressed in neutrophils, in contrast to MPs where HIF-1α is the most active. HIF-2α deficiency increases neutrophil apoptosis (71). MIP-1 is also identified as a novel hypoxia stimulated granulocyte survival factor (72).

In the ZF model infected with Mm, neutrophil-specific HIF-1α stabilization decreases bacterial burden via a NO-dependent mechanism. On the contrary, despite also being upregulated, HIF-2α has a negative impact on bacterial burden emphasizing opposite roles of different HIF factors (73). Therefore, it is possible that the hypoxic environment of the TB granuloma that favors extended life-span for neutrophils allows them to actively shape granuloma evolution. On one hand, this may help bacilli control as well as resolution of inflammation, since neutrophils actively participate to MP efferocytosis and the release of lipids such as LXA4. On the other hand, hypoxia increases neutrophil degranulation and confers extended activity to damage lung tissues in a PI3K dependent pathway (74). Hypoxia-induced decrease of neutrophil apoptosis induces a delay in resolution of inflammation by maintaining active neutrophils in the inflamed tissue (75). Moreover, hypoxia impairs the ability of neutrophils to kill certain bacteria (76).

HIF-1α is a major player in an another chronic infection caused by the intracellular parasite Leishmania. HIF-1α crucially enhances immunosuppressive functions of MDSCs and decreases leishmanicidal capacity of myeloid cells (77). Even though to our knowledge no study has tackled the link between HIF-1α and MDSCs in TB, a similar important role could be discovered. Also, since hypoxia and angiogenesis are intimately linked to the granuloma development, another interesting perspective is the possible role of angiogenic neutrophils (40, 42) in the process.

## Possible Influence of the Hypoxic Mtb Granuloma on Key Neutrophil-Released Mediators

**Figure 1:**
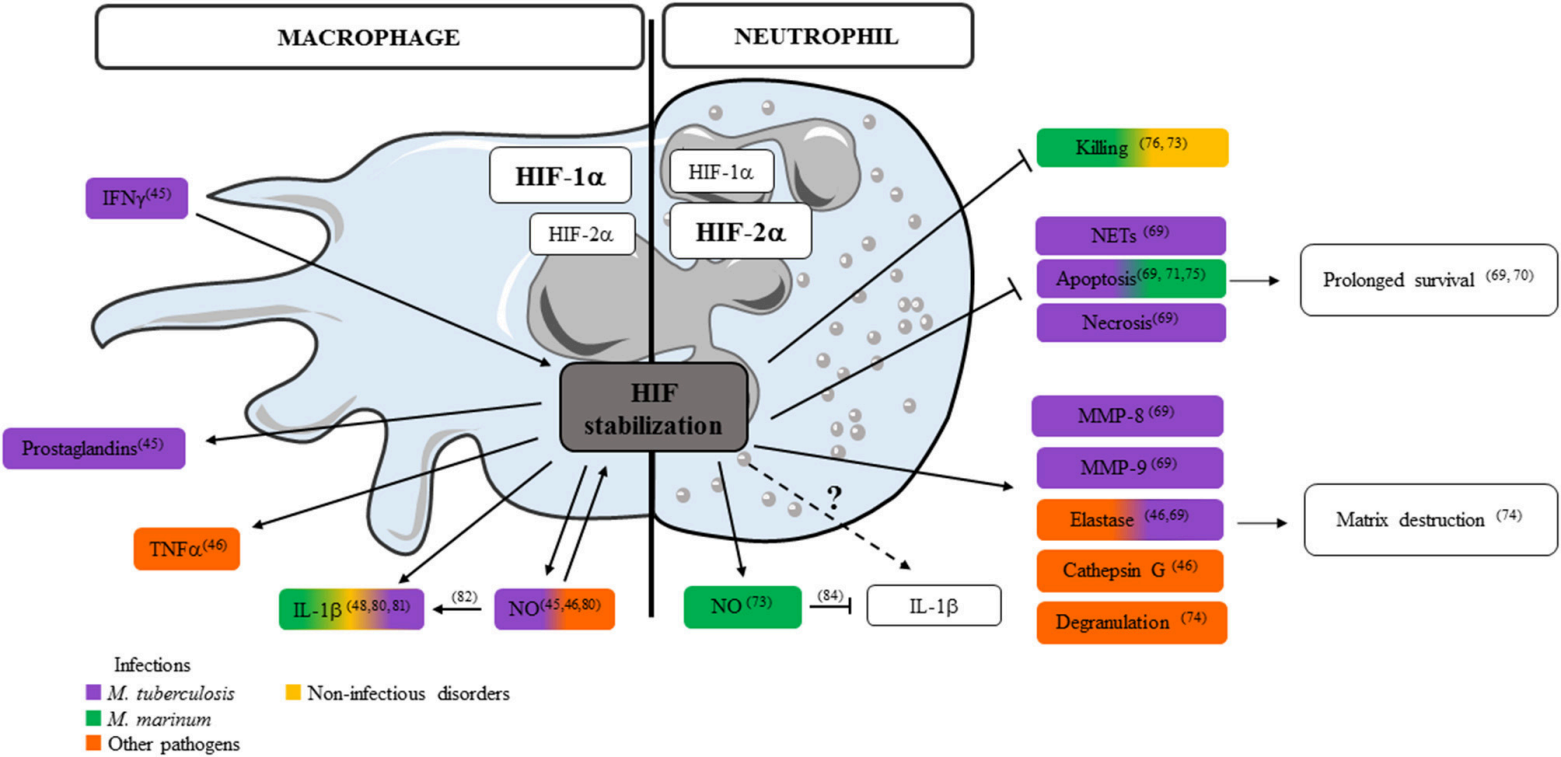
Impact of HIFs on control of key mediators released by neutrophils and macrophages. Key mediators and essential cellular processes controlled by HIF stabilization in macrophage (left) or neutrophil (right) after infections with *M. tuberculosis, M. marinum*, or other bacteria or during non-infectious disorders are depicted (numbers refer to publications listed in the review).

Neutrophils contribute both pro- and anti-inflammatory factors in TB (78, 79). Information on how HIF-1α stabilization in hypoxic environment influences the secretion of critical immune mediators by neutrophils is limited to granule proteases, antimicrobial peptides and TNF (46). Literature on the impact of HIF-1α stabilization on MP-released mediators is more extensive and we consider it as a source of inspiration illustrating the potential role of hypoxia on neutrophil-released mediators (Figure 1). In the following paragraph, we focus on how hypoxia may regulate release by neutrophils of the key mediators in Mtb virulence: ROS, NO, IL-1β, and type I IFN. Some of these mediators may play different roles in humans and animal models and data should sometimes be interpreted with caution. In Mtb infected MPs, HIF-1α is stabilized by IFN-γ and regulates the production of prostaglandins and NO (45). In mice, NO not only acts as an antimicrobial agent and inflammatory mediator but further amplifies myeloid cell bactericidal activity via HIF-1α stabilization. NO modulates the MP response to Mtb through activation of HIF-1α and repression of NF-kB (80). HIF-1α and NO are intrinsically linked: they positively regulate each other, but display distinct roles in the regulation of inflammation. Among the mediators regulated in opposite directions, neutrophil-attracting chemokines, IL-1α and IL-1β, are all down regulated in HIF-1α^−/−^ and upregulated in Nos2^−/−^ Mtb infected and IFN-γ activated BMDMs (80). In the hypoxic granuloma, NO produced by IFNγ-activated MPs restricts neutrophil recruitment to avoid destructive inflammation (80). In Mm infected ZF, HIF-1α stabilization induced IL-1β production by MPs and increased neutrophil NO production that is protective against infection (81). In Mm infected NADPH oxidase 2-deficient mice (Ncf1^−/−^) mice, ROS-deficiency decreases IL-1β production by MPs but induces early and extensive neutrophilic inflammation, with high elastase activity and IL-1β production (82). This also reveals a novel role for ROS in the early neutrophilic granulomatous inflammation and the importance of neutrophil-driven IL-1β production during mycobacterial infection. In addition to MPs, neutrophils also produce ROS and NOS. Neutrophils are able to discriminate pathogens by differential production and localization of ROS, and tune their own recruitment and distribution to exquisitely tailor the anti-microbial response (83). HIF-1α stabilization in neutrophils induces NO production after infection by Mm (73). NOS and ROS production also influences the secretion of cytokines. NO inhibits NLRP3-dependent IL-1 responses (84). IL-1β signaling is also important for ROS production as Mtb-infected newly recruited neutrophils lacking IL-1R fail to produce ROS, resulting in compromised pathogen control (85). HIF-1α stabilization clearly influences ROS/NOS and IL-1β production by MPs and neutrophils, both factors are important during mycobacterial infection, but their regulation seems different in the two cell types (73, 80–82, 84, 85) (Figure 1). In human neutrophils stimulated with Mtb, hypoxia up-regulates secretion of MMP-8, MMP-9 and neutrophil elastase that are involved in matrix destruction. Hypoxia inhibits NETs formation and both neutrophil apoptosis and necrosis after direct stimulation by Mtb (69).

Type I IFN is a major cytokine in TB pathophysiology. Overproduction of type I IFN (IFN-I) is linked to exacerbated TB in both mouse models and humans. IFN-I triggers immunopathology by increasing the recruitment of inflammatory monocytes and neutrophils to the lung (86). Secretion of IFN-I by MPs and its effect on neutrophils is well-documented (87–89). In MPs, Mtb triggers IFN-I secretion through bacterial DNA release in the cytosol. However, strains display variable ability to activate the IFN-I pathway depending on their effective triggering of mitochondrial stress (87). Host-protective cytokines such as TNF, IL-12, and IL-1β are inhibited by exogenous IFN-I, via production of immunosuppressive IL-10 (88). By contrast, IL-1β suppresses IFN-I through eicosanoid prostaglandin E2 (90). In the inflammatory environment in mouse tumor models, IFN-I shifts neutrophils to antimetastastic phenotype (89). Therefore, IFN-I has multiple and crucial effects on neutrophils, but so far studies on IFN signaling in neutrophils in hypoxic environment are still scarce. As hypoxia leads to accumulation of cytosolic DNA via mitochondrial or nuclear DNA damage, it could favor activation of the cGAS/STING/IRF3 pathway (91). The convergence between hypoxia and IFN-I signaling is suggested by Karuppagounder et al. who identified the effect of Tilorone, a small molecule inducing IFN-I which also triggered hypoxic response in brain cells (91). Another study claims that IFN-I promotes tumorigenic properties through up-regulation of HIF functions in different cancer cell lines (92). Direct IFN-I secretion by neutrophils is proposed, where Sox2 could act as a sequence-specific DNA sensor in neutrophils during microbial infection (93). However, it is unclear if neutrophils can sense DNA via the cGAS/STING pathway (94).

Thus, even though the impact of hypoxic environment encountered in the TB granuloma on the IFN-I pathway is not documented yet, this issue could be of great interest to better understand TB pathophysiology and propose new therapies.

## Neutrophils in TB: Many Open Questions

Although it is now consensus that during active TB, neutrophils are the main villains responsible for lung destruction, we–and others (3, 11)–advocate that this narrow vision is revisited. “Neutrophils” encompass different subsets with various functions that remain poorly defined. They come to infectious foci in waves of different magnitude. A better definition of neutrophil subsets, their coordinated dynamics of recruitment to the lung and their associated functions is needed. Neutrophils crosstalk with other cells and secrete a vast number of mediators thus taking full part to the regulation of the immune response against Mtb. They respond to signals sent by their environment, including hypoxia in inflamed tissues. In the hypoxic TB granuloma, light has been recently shed on the fate and behavior of MPs, under the control of the master regulator HIF-1α. However, there is currently scarce information on the fate and behavior of neutrophils in a similar context. How do neutrophils respond to hypoxia in the TB context? How do neutrophils contribute to the shaping of the granuloma? In the future, models allowing development of the hypoxic TB granuloma should be favored. A better definition of mediators released by neutrophils in the hypoxic context of the granuloma is expected. As neutrophils are key players in the game, we believe that these questions need to be solved in order to propose new interventions to fight against TB.

## Perspectives for Innovative Therapeutics Against TB

In the era of increasing multidrug resistance of Mtb strains, HDTs sometimes represent the last hope for patients. As the hallmark of destructive inflammation, neutrophils are often considered as potential targets. Inhibiting necrotic neutrophil death could restore Mtb growth control (24). Removing neutrophils by drugs or immunotherapeutic interventions could also alleviate lung tissue destruction.

Recent studies in the TB field shed some light on parallels that could be drawn between the TB granuloma and solid tumors (Table 1), especially regarding the role of neutrophils. Since HDTs are more advanced in the field of cancer than they are in TB, we propose that some tracks well-developed in cancer therapy are explored to advance the field of HDTs for TB patients. Among promising avenues, we propose that metabolic changes occurring in TB granuloma are being considered (113). Modulation of the HIF pathways (114) deserves attention as it could dampen excessive protease secretion (69). PHD3 and HIF-2α that operate in neutrophils under inflammatory or hypoxic conditions (70, 71) represent more attractive targets than largely distributed HIF-1α. In cancer, another active field in the clinics is blocking angiogenesis since this pathway is key to tumor development. Angiogenesis appeared more recently as key to the development of the TB granuloma and it would be interesting to determine whether modulating angiogenesis could bring some benefit to TB patients. Along this line, we believe that recently described angiogenic neutrophils should also be investigated in TB.

**Table 1:** Impact of neutrophils and close relatives in cancer and TB.

	**Tumor formation and evolution**	**Early Mtb infection and granuloma**
Prognosis/Pathophysiology	• Clinical evidence (neutrophil to lymphocyte ratio) mostly links neutrophils to cancer progression. Poor prognosis. • Different granulocytic populations described with various functions. Anti-tumor activity of High Density Neutrophils (early stage tumor). Accumulation of immature neutrophils associated to cancer progression (Gr MDSCs or Low Density Neutrophils). Promote angiogenesis, tumor progression, and metastases (95) • In many cancers, different granulocyte subpopulations are described (96). Better definition is needed	• Early phase of infection: neutrophils contribute to innate resistance (11, 97) and granuloma formation (7, 98, 99) • Late phase of infection, active TB: established role of neutrophils to severe forms in preclinical models (8) and in humans (9) • Gr-MDSCs accumulate during early phase Mtb infection and active TB, in blood and lung in humans (31) • MDSCs may represent permissive reservoir for Mtb (34) and their accumulation associates with severe TB in mice (35)
Hypoxia and angiogenesis	• HIF-2α, selectively modulates neutrophil recruitment (100) • Neutrophil recruitment to early-stage tumors is linked to hypoxia (101) • Induction of angiogenic neutrophils in hypoxic conditions	• Hypoxia augments neutrophil degranulation and confers enhanced potential for damage to respiratory airway epithelial cells (69) • Hif-1α increases NO production by neutrophils in early stage of Mm infection and is involved in control of bacterial growth (73) • Granuloma formation in the ZF model coincides with angiogenesis and local hypoxia (62)
Modulation of T cell response	• MDSCs are major players in tumor-mediated immunosuppression • Neutrophils in solid tumors are potent producers of Arg-1 and could contribute to local immune suppression (102, 103)	• MDSCs are present in lungs (3) but their role in development and evolution of granuloma remains unclear • Arg-1 is associated to severe TB in mouse models (104, 105) and is detected in necrotizing granulomas in humans (106). Pathway documented in MPs, however, deciphering neutrophil contribution to Arg-1 production would be of interest.
Tissue Remodeling	• MMP-9 delivered by tumor-recruited neutrophils is associated to tumor angiogenesis and dissemination (107) • Angiogenic neutrophils contribute to tumor growth and metastasis (108) • Neutrophils through COX-2-mediated PGE2 synthesis and elastase promote tumor cell proliferation (109)	• MMPs are involved in early granuloma formation and participate to tissue destruction during late phase (110, 111) • Pathogenic mycobacteria (Mm or Mtb) exploit the formation of new blood vessels to disseminate via MPs (62, 63). Neutrophils are migrating cells (112), their contribution to Mtb dissemination is not documented yet.

TB still kills 1.7 million people each year and active TB patients continuously spread bacilli that represent threat to human kind. Development of adjunct HDTs is a promising avenue to boost current drug regimen directed against Mtb (115). We believe that addressing the questions that we raised in this review about neutrophils in TB could greatly help in the quest for innovative HDTs.

## Author Contributions

All authors listed have made a substantial, direct and intellectual contribution to the work, and approved it for publication.

### Conflict of Interest Statement

The authors declare that the research was conducted in the absence of any commercial or financial relationships that could be construed as a potential conflict of interest.

## Footnotes

^1^http://www.who.int/gho/tb/en/
